# Commentary: Overweight and Cognitive Performance: High Body Mass Index Is Associated with Impairment in Reactive Control during Task Switching

**DOI:** 10.3389/fnut.2019.00198

**Published:** 2020-01-31

**Authors:** Frontiers Editorial Office

**Affiliations:** Frontiers Media SA, Lausanne, Switzerland

**Keywords:** cognitive flexibility, adiposity, overweight, body mass index, task switching, binge eating

With this notice, Frontiers states its awareness of major concerns regarding the data underlying this study. These concerns have been communicated to the publisher by the institution of the authors at the time of publication, the University of Leiden. This expression of concern has been posted while Frontiers investigates the data concerns.

## Conflict of Interest

The authors declare that the research was conducted in the absence of any commercial or financial relationships that could be construed as a potential conflict of interest.

